# Enhancing feedback on performance measures: the difference in outlier detection using a binary versus continuous outcome funnel plot and implications for quality improvement

**DOI:** 10.1136/bmjqs-2019-009929

**Published:** 2020-02-07

**Authors:** Laurien Kuhrij, Erik van Zwet, Renske van den Berg-Vos, Paul Nederkoorn, Perla J Marang-van de Mheen

**Affiliations:** 1 Department of Neurology, Amsterdam University Medical Centres, Amsterdam, The Netherlands; 2 Department of Biomedical Data Sciences, Leiden University Medical Center, Leiden, Zuid-Holland, The Netherlands; 3 Department of Neurology, OLVG, Amsterdam, Noord-Holland, The Netherlands

**Keywords:** audit and feedback, quality improvement, performance measures

## Abstract

**Background:**

Hospitals and providers receive feedback information on how their performance compares with others, often using funnel plots to detect outliers. These funnel plots typically use binary outcomes, and continuous variables are dichotomised to fit this format. However, information is lost using a binary measure, which is only sensitive to detect differences in higher values (the tail) rather than the entire distribution. This study therefore aims to investigate whether different outlier hospitals are identified when using a funnel plot for a binary vs a continuous outcome. This is relevant for hospitals with suboptimal performance to decide whether performance can be improved by targeting processes for all patients or a subgroup with higher values.

**Methods:**

We examined the door-to-needle time (DNT) of all (6080) patients with acute ischaemic stroke treated with intravenous thrombolysis in 65 hospitals in 2017, registered in the Dutch Acute Stroke Audit. We compared outlier hospitals in two funnel plots: the median DNT versus the proportion of patients with substantially delayed DNT (above the 90th percentile (P90)), whether these were the same or different hospitals. Two sensitivity analyses were performed using the proportion above the median and a continuous P90 funnel plot.

**Results:**

The median DNT was 24 min and P90 was 50 min. In the binary funnel plot for the proportion of patients above P90, 58 hospitals had average performance, whereas in the funnel plot around the median 14 of these hospitals had significantly higher median DNT (24%). These hospitals can likely improve their DNT by focusing on care processes for all patients, not shown by the binary outcome funnel plot. Similar results were shown in sensitivity analyses.

**Conclusion:**

Using funnel plots for continuous versus binary outcomes identify different outlier hospitals, which may enhance hospital feedback to direct more targeted improvement initiatives.

## Introduction

Hospitals and providers receive feedback on the quality of care delivered in many ways, for example, in feedback from patients on their experiences and in how the outcomes and care processes for these patients relate to those achieved in other hospitals. Hospitals and providers typically use such feedback information to see which processes and outcomes need to be improved and try to identify which patient groups should be targeted for these quality improvement initiatives.

Funnel plots are often used in feedback information to identify outliers, either comparing the performance across hospitals or providers to identify those with significantly better or worse performance.^1 2^ Based on their position in the funnel plot, particularly when indicating significantly worse performance, hospitals or providers search for explanations on how and where to improve care. Historically, these funnel plots were typically applied for binary outcomes, such as mortality or complication rates after surgery, usually with case-mix adjustment for fair comparison.^3^ Hereafter, perhaps because of the familiarity with this type of funnel plots, continuous outcomes were often converted to binary outcomes according to a norm or performance threshold rather than using the continuous values. An example of a quality indicator that is dichotomised is the door-to-needle time (DNT) in patients who had acute cerebral ischaemic stroke (AIS), where the Stroke Early Management Guidelines by the American Hearts Association/American Stroke Association stated that at least 50% of all patients need to have a DNT of 60 min or less.^4^ Another example where funnel plots are used to compare hospitals is the DNT in patients with acute myocardial infarction treated with thrombolysis using a cut-off point of 30 min.^5^

However, by using cut-off points for continuous variables to create a binary outcome, important information is lost as only the tail of the distribution in the study population is investigated (ie, patients with higher values), rather than the entire distribution. This missing information could help a hospital to answer the question whether their average performance can be improved by improving a little for all patients, that is, focusing on processes of care affecting all patients or to focus on a subpopulation of patients with higher values and target care processes particularly relevant for these patients. For instance, in time to treatment, delay could be taking place in the entire population, implying that all patients are experiencing some form of delay. However, such a hospital does not have to be an outlier in a funnel plot with time to treatment dichotomised as a binary outcome, as this funnel plot is sensitive to detect whether a hospital has significantly more patients with long delays (the tail of the distribution). However, if average performance is suboptimal because in fact only a small group of patients experience long delays, this will be detected in a funnel plot on binary outcomes and enable such a hospital to initiate improvement initiatives for this group specifically. Currently, it is not possible to differentiate between these two situations as this would require a different type of funnel plot, sensitive to detect differences in the entire distribution.

The aim of this study is to investigate whether different hospitals would be identified as outliers when using two different funnel plots: a funnel plot for a binary outcome and one with a continuous outcome. This is illustrated using DNT in patients who had AIS treated with intravenous thrombolysis (IVT) in the Netherlands as an example, using data from the nationwide Dutch Acute Stroke Audit (DASA).^6^ Given the short median DNT in the Netherlands compared with other countries,^7 8^ one could argue that further improvements are most efficiently achieved by reducing the DNT for patients with substantial delays and that hospitals with many of those patients will be the same hospitals as those with high median DNT.

## Methods

### Data source and patient selection

All patients with AIS treated with IVT in 2017 in the Netherlands and registered in the DASA were included. The DASA is a nationwide, registry-based, prospective clinical audit in which data are collected from patients with AIS and haemorrhagic stroke since 2014.^6^ The DASA is managed by the Dutch Society of Neurology, a nationwide professional organisation for neurologists, and facilitated by the Dutch Institute for Clinical Auditing (DICA). It uses indicators to measure quality of care and provides a national benchmark including DNT.

The median DNT is an important and widely acknowledged indicator of quality of care and used in auditing and hospital performance comparisons in the Netherlands^8^ as well as in many other countries.^9^ The DNT is desired to be as short as possible as the effect of IVT is strongly time dependent.^10^ However, there are a few contraindications to IVT, some of which are reversible and need to be treated before IVT is administered, such as hypertension, emergent medical conditions (for instance seizures or respiratory insufficiency) or inability to determine time of onset of symptoms from the patient. Therefore, a delay in DNT is sometimes unavoidable in a small group of patients to provide good stroke care, for instance to take time to lower the blood pressure in case of hypertension or to contact next of kin to make a better estimation of time of onset.^11 12^ This may be among the explanations for a hospital having a higher median DNT, if they have a high proportion of patients with substantial delays.

### Definitions

DNT was defined as the difference between door time and needle time in minutes. The door time was defined as the time of presentation at the emergency room with a stroke. In case the patient was already admitted to the hospital (ie, in-hospital stroke), the time of examination by the neurologist was used as the door time. Whether the patient experienced an in-hospital stroke is not registered separately in the DASA, so the data only include the door time without specifying whether the stroke occurred in hospital or in the community. Needle time was defined as the time when the (bolus of) IVT was given. Provided that the number of patients with unknown DNT was below 5% of all patients, these patients were excluded from analysis. A substantial delay in DNT was defined as a DNT higher than the 90th percentile, which was 50 min.

Other variables used to characterise differences in hospitals or patients with or without delayed DNT were: age, sex, National Institutes of Health Stroke Scale (NIHSS) score (a measure for the severity of stroke ranging from 0 to 42 with higher scores indicating more impairment for the patient), admission during off-hours and admission at a comprehensive stroke centre. Admission during off-hours was defined as admission outside office hours from Monday to Friday, that is, between 18:00 and 08:00 hours, or on Saturday or Sunday. A comprehensive stroke centre was defined as a hospital that is certified to perform intra-arterial thrombectomy.

### Statistical analysis

Baseline patient characteristics (age, sex, NIHSS score, admission during off-hours and admission at a comprehensive stroke centre) were compared for patients above and below the 90th percentile, using χ^2^ tests for categorical variables and the t-test for continuous variables. Continuous values were expressed as mean with SD and nominal variables as count and percentages. Time intervals as well as the NIHSS score were not normally distributed and therefore summarised by median and corresponding IQRs. The Kruskal-Wallis test was used to test for differences between groups in these variables.

Next, two types of funnel plots were created with 95% control limits, with hospitals outside these limits performing statistically significantly different from the nationwide value (outliers, indicating special cause variation). The first funnel plot is created for the binary outcome, the proportion of patients for each hospital with a DNT above the 90th percentile (50 min). This funnel plot demonstrates whether hospitals have higher, similar or lower proportion of patients with a substantial delay (ie, the tail of the distribution) compared with the nationwide proportion. The second funnel plot is created for the continuous outcome, comparing the median DNT of each hospital to the nationwide median DNT (24 min). This funnel plot typically detects whether the entire distribution is different and potentially shifted, in other words whether DNT is higher for all patients with AIS for whom IVT is indicated. The choice for this combination of funnel plots was based on hospitals already being familiar with the binary funnel plots and its interpretation, with the 90th percentile reflecting patients with substantially delayed DNT likely to complement the information from the median DNT, which is also a familiar statistic for most professionals in stroke care.

In both funnel plots, hospitals above the upper control limit with worse performance are coloured red. Hospitals below the lower control limit with better performance are coloured blue. Hospitals between control limits are coloured grey. Formulas used to calculate the funnel plots are shown in online supplementary file 1 1. To combine both funnel plots in evaluating the performance for each hospital and to show the added value of the novel funnel plot for continuous outcomes, hospitals are coloured given their position in the funnel plot around the median and visualised in that colour in the funnel plot for the proportion of patients with a substantially delayed DNT.

10.1136/bmjqs-2019-009929.supp1 Supplementary data

In addition, each hospital was classified as below the lower control limit, within control limits or above the upper control limit for both funnel plots to assess the extent to which hospitals are classified differently depending on the type of funnel plot. For hospitals with a significantly higher median DNT, we compared characteristics of patients treated in hospitals that also had a significantly higher proportion of patients with substantially delayed DNT with patients treated in hospitals having a similar proportion of these patients than the nationwide average.

Last, we performed two sensitivity analyses. In the first sensitivity analysis, rather than using the extreme tail of the distribution for the 90th percentile to create the binary outcome, we created a binary outcome funnel plot for the proportion of patients with a DNT higher than the median to be compared with the continuous outcome funnel plot around the median. Given that the median is now used in both funnel plots, comparing a continuous measure containing more information (and thus more power) with a binary measure, this analysis shows how creating binary outcomes affects hospitals being identified as outliers. In the second sensitivity analysis, we compared two continuous outcome funnel plots, one around the median with one around the 90th percentile DNT. Given that both funnel plots use continuous outcomes, containing most information, this analysis shows how different hospitals may be identified as outliers depending on which part of the distribution of DNT is different (the tail or entire distribution) without the possibility that part of the difference is being caused by using a binary versus a continuous outcome.

R studio version 3.4.3 was used for statistical analysis.

## Results

**Figure 1:**
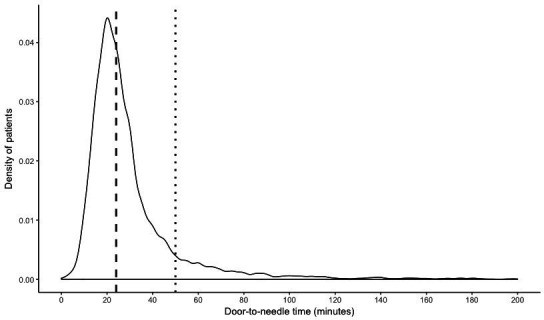
Density plot of DNT. The nationwide median DNT is shown as the dashed line and the 90th percentile of DNT as dotted line. DNT, door-to-needle time.

In 2017, 6185 patients with AIS from 65 different hospitals were treated with IVT. One hundred and five patients (1.7%) were excluded due to missing DNT time. Therefore, 6080 patients were included for analysis. The nationwide median DNT of all patients was 24 min. The nationwide 90th percentile of DNT, that is, the patients that had a substantial delay to IVT, was 50 min. The distribution of DNT for all Dutch patients is shown in figure 1.

The baseline characteristics for all patients are shown in table 1. The mean age was 71 years, and 46% was female. The median NIHSS score was 5 (IQR 3–9). In patients with a DNT above the 90th percentile, the median NIHSS score was 4 (IQR 2–8). This is significantly lower than the median of 5 in patients with a DNT below the 90th percentile (p<0.001), although the difference of 1 point in NIHSS score is not clinically relevant. Thirty-five per cent of the patients had an unknown NIHSS. It is difficult to assess whether these are missing at random, particularly since there were significantly more unknown NIHSS scores in patients with a DNT above the 90th percentile (p=0.01). Forty-nine per cent of patients were admitted during off-hours. Forty-one per cent (2502 patients) were admitted to 1 of the 18 comprehensive stroke centres in the Netherlands.

**Table 1:** Baseline characteristics for all patients and differences between patients with and without a substantially delayed DNT indicated by the 90th percentile

	Total no of patients n=6080	Patients with DNT ≤90th percentile n=5478	Patients with DNT >90th percentile n=602	P value
Mean age in years (SD)	71 (13)	71 (13)	71 (14)	0.46
Female sex (%)	46	46	49	0.19
Median NIHSS* score (IQR)	5 (3–9)	5 (3–10)	4 (2–8)	<0.001
Unknown NIHSS score (%)	35	35	40	0.01
Admission during off-hours (%)	49	49	48	0.46
Admission at a comprehensive stroke centre (%)	41	41	43	0.30

P value represents statistical difference between dichotomised groups.

*NIHSS score.

DNT, door-to-needle time; NIHSS, National Institutes of Health Stroke Scale.

**Figure 2:**
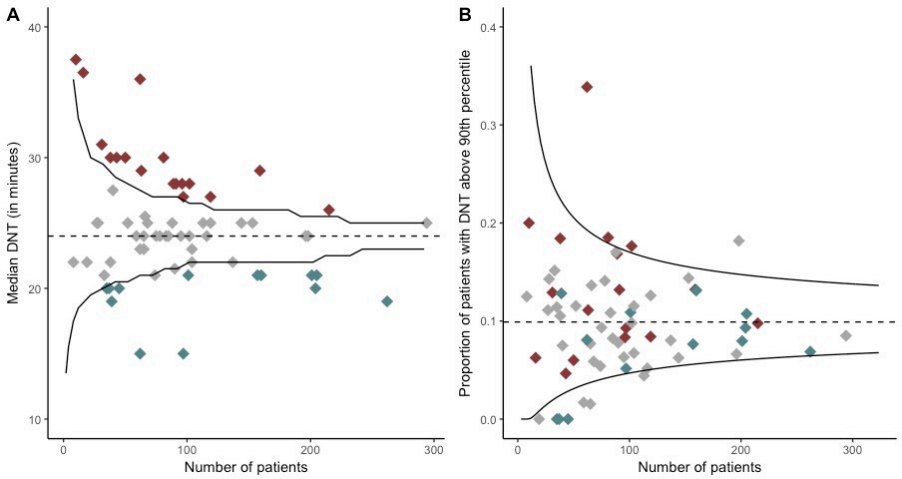
(A) The median DNT for each hospital and the number of patients treated at each hospital is shown. Each diamond represents one hospital. (B) The proportion of patients with a substantially delayed DNT (indicated by the 90th percentile) and the number of patients treated at each hospital is shown. Each diamond represents one hospital. The black lines are the 95% control limits. The red diamonds are hospitals with statistically significantly longer median DNT than the nationwide median. The grey diamonds are hospitals with a similar median DNT. The blue diamonds are hospitals with a statistically significantly shorter median DNT than the nationwide median. DNT, door-to-needle time.

In figure 2A, hospitals are evaluated based on their median DNT in a funnel plot. Seventeen hospitals had a median DNT above the upper control limit and therefore a longer DNT than the nationwide median of 24 min. In these 17 hospitals, 1362 patients (22%) were treated. Thirteen hospitals had a shorter median DNT, treating 1605 patients (26%). As shown in figure 2B, four hospitals had a significantly higher proportion of patients with a substantially delayed DNT. Seven hospitals had a lower proportion of patients with a substantially delayed DNT, of which four hospitals did not have any patients with a substantially delayed DNT.

In the same figure, it is shown by the colours how the hospitals distribute when looking at substantial delay in DNT given their status on the median DNT. Table 2 also shows this distribution. Out of the 13 hospitals with a shorter median DNT, three hospitals had no patients with a substantially delayed DNT. The remaining 10 hospitals had a proportion of patients with a substantial delay similar to the nationwide average. Out of the 17 hospitals with a significantly longer median DNT, 14 hospitals had a proportion of patients with a substantial delay similar to the nationwide average. The remaining three hospitals, which treated 245 patients, also had a higher proportion of patients with a substantially delayed DNT. Two of these three hospitals were comprehensive stroke centres. Among the patients treated in these three hospitals, the median NIHSS was higher (7 vs 5), patients were younger (68 vs 71 years) and more often female (53% vs 43%) than in the 14 hospitals having a higher median DNT combined with a similar proportion of substantially delayed patients with similar rate of admission during off-hours (51% vs 50%). Had the performance been evaluated looking at the proportion of patients with substantially delayed DNT, 58 hospitals would have considered to have no need for improvement, whereas in fact 14 (24%) of those had a significantly longer median DNT than the nationwide median. The combination of the two funnel plots thus gives complementary information and gives direction which patients should be targeted for initiatives to improve DNT, as summarised in table 3.

**Table 2:** Distribution of hospitals in funnel plot around the median DNT versus the funnel plot with the proportion of patients with a DNT above the 90th percentile

		Position of hospital in funnel plot around the median DNT (no. of hospitals)			
		Below lower control limit	Between control limits	Above upper control limit	Total
Position of hospital in funnel plot with proportion above 90th percentile (no. of hospitals)	Below lower control limit	3	4	0	7
	Between control limits	10	30	14	58
	Above upper control limit	0	1	3	4
	Total	13	35	17	65

DNT, door-to-needle time.

**Table 3:** Summary of complementary information given by the combination of funnel plots and how this might be used to inform quality improvement strategies

		Position of hospital in funnel plot around the median DNT	
		Within control limits	Above upper control limit
Position of hospital in funnel plot with proportion above 90th percentile	Within control limits	No immediate actions needed.	Performance on DNT might be improved by targeting processes affecting all patients.
	Above upper control limit	Performance on DNT might be improved by investigating which factors are causing delays in the subpopulation of patients with substantial delay. This might result in further reduction of median DNT.	Improvement strategy depends on whether the shape of the DNT distribution is: (1) similar as nationwide or (2) skewed and stretched towards substantial delays. The first suggests the entire distribution may have shifted to higher values and that improvement on DNT might be obtained by targeting processes of all patients. The second suggests that the subpopulation of patients with substantial delay may be causing the higher median and that these patients should be targeted to examine which factors might be causing the delay to improve performance on DNT.

DNT, door-to-needle time.

**Figure 3:**
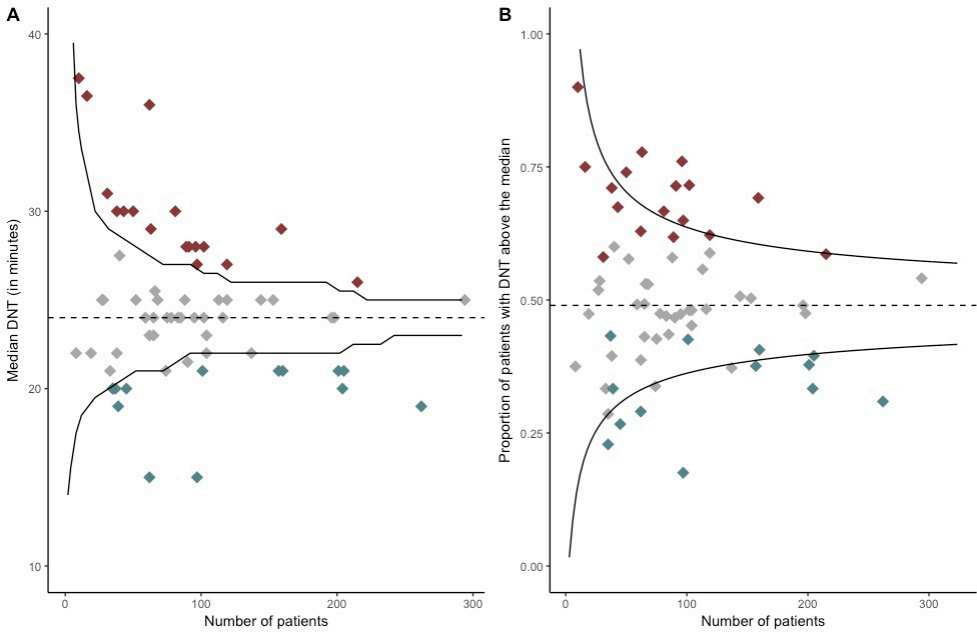
(A) The median DNT for each hospital and the number of patients treated at each hospital is shown. Each diamond represents one hospital. (B) The proportion of patients with a DNT above the median and the number of patients treated at each hospital is shown. Each diamond represents one hospital. The black lines are the 95% control limits. The red diamonds are hospitals with statistically significantly longer median DNT than the nationwide median. The grey diamonds are hospitals with a similar median DNT. The blue diamonds are hospitals with a statistically significantly shorter median DNT than the nationwide median. DNT, door-to-needle time.

Comparing the funnel plots for median DNT and the proportion of patients with a DNT above the median illustrates that fewer hospitals are identified with worse performance when using a funnel plot for a binary outcome (figure 3). Out of the 17 hospitals with a significantly higher median DNT, only eight hospitals also had a higher proportion of patients with a DNT above the median (table 4). Out of the 13 hospitals with a lower median DNT, eight hospitals also had lower proportion of patients with a DNT above the median. If the performance had solely been evaluated based on the binary outcome, then 47 hospitals would have considered no need for improvement given a similar proportion of patients above the median, whereas in fact nine (19%) of these hospitals had a median DNT significantly worse than the nationwide median. By creating a binary outcome funnel plot, fewer hospitals are thus identified as outliers either with better or with worse performance.

**Table 4:** Distribution of hospitals in funnel plot around the median DNT versus the funnel plot with the proportion of patients with a DNT above the median

		Position of hospital in funnel plot around the median DNT (no. of hospitals)			
		Below lower control limit	Between control limits	Above upper control limit	Total
Position of hospital in funnel plot with proportion above the median DNT (no. of hospitals)	Below lower control limit	8	2	0	10
	Between control limits	5	33	9	47
	Above upper control limit	0	0	8	8
	Total	13	35	17	65

DNT, door-to-needle time.

**Figure 4:**
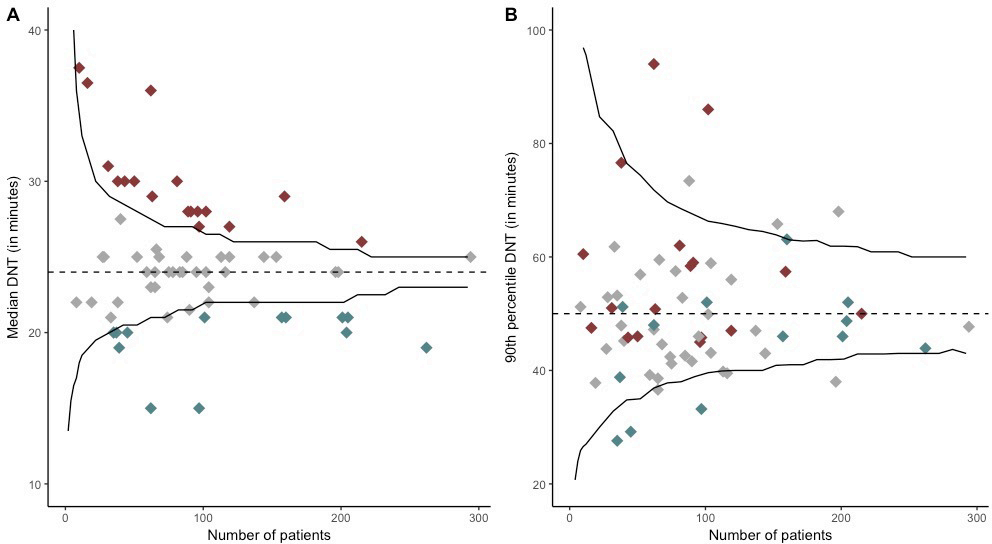
(A) The median DNT for each hospital and the number of patients treated at each hospital is shown. Each diamond represents one hospital. (B) The 90th percentile of the DNT for each hospital and the number of patients treated at each hospital is shown. Each diamond represents one hospital. The black lines are the 95% control limits. The red diamonds are hospitals with statistically significantly longer median DNT than the nationwide median. The grey diamonds are hospitals with a similar median DNT. The blue diamonds are hospitals with a statistically significantly shorter median DNT than the nationwide median. DNT, door-to-needle time.

Additionally, comparing two funnel plots with a continuous outcome, similar results are shown as in our baseline analysis except that two rather than three of the hospitals with significantly higher median DNT also had a higher 90th percentile DNT (figure 4).

## Discussion

This study has shown that the commonly used funnel plot for a binary outcome identifies different hospitals as having worse performance than using a funnel plot for a continuous outcome. In the example used in this study, most hospitals with a significantly longer median DNT did not have a higher proportion of patients with substantially delayed DNT. And the other way around, 14 out of 58 hospitals with a similar proportion of patients with substantially delayed DNT have a significantly longer DNT than the nationwide median and thus potentially in scope for further improvement. Both funnel plots provide different information and are important for hospitals to identify where to target improvement initiatives. Dependent on the position in the funnel plots, this might involve the improvement of processes for all patients or the investigation of the group of patients with substantially delayed DNT on possible explanatory factors and need for improvement in care processes relevant for this subgroup.

The idea of improvement needed for the entire population or a specific group with higher values at the tail of the distribution is not new. In the context of prevention strategies, it has been described as the prevention paradox: shifting the entire distribution a little versus targeting only high-risk patients. Shifting the entire distribution, the effect for the individual is small but is significant for the entire population, whereas targeting high-risk patients only will result in large benefits for the individuals but potentially small population effects.^13^ Findings in other studies include for instance the beneficial effect of statins on risk of ischaemic heart disease and/or stroke in patients with high cholesterol.^14^

In our example of patients with AIS, a small effect for the individual could be important, as the effect of IVT is time dependent and the patient should be treated as soon as possible after stroke onset to increase the odds of a good outcome. In practice, however, many patients are still too late to be eligible for reperfusion therapy such as IVT. By reducing DNT further if only by a small amount given the low nationwide median, more patients could be eligible for IVT, which might increase thrombolysis rates and thus very relevant information for hospitals as feedback. However, an unintended consequence of reducing the DNT further can be that more ‘stroke mimics’, which are non-vascular disorders with symptoms resembling stroke, are treated with IVT.^15^ Given that multiple studies have shown IVT treatment to be safe for patients with stroke mimics, this does not have to interfere with administering IVT as soon as possible.^16 17^

Therefore, the aim should be to reduce the DNT as much as possible. To achieve this, it is important for hospitals to get feedback on their median DNT being significantly worse to inform them that they may be able to improve the general processes for all patients, even though they do not have a high percentage of patients with substantially delayed DNT. Similarly, if a hospital is not an outlier on the median DNT but has significantly more patients with substantially delayed DNT, this information may direct a hospital to investigate those patients with substantially delayed DNT on potential explanatory factors and whether there are aspects that can be improved for these patients. Some patients have justified substantial delay due to reversible contraindications that should be taken into account when evaluating quality of care by the duration of the DNT. In this study, we assumed that patients with a substantial delay are those with a DNT above the 90th percentile, which is 50 min and thus closer to the 60 min used in international guidelines,^4^ but other cut-off points could be argued (for instance the 75th percentile). Additionally, we performed two sensitivity analyses. In the first sensitivity analysis, we used the proportion around the median, which showed that fewer hospitals would be identified as outlier when dichotomising a continuous variable, showing the higher power for the funnel plot with a continuous outcome to detect outliers. In the second sensitivity analysis, we used continuous outcomes for both funnel plots showing similar results as in the baseline analysis. The slight difference might be caused by the continuous funnel plot for the 90th percentile having low power, as it is more difficult to precisely estimate such an extreme percentile. We therefore advise to use the combination of a funnel plot around the median with a funnel plot for the proportion of patients above the 90th percentile, to be used as complementary information for giving feedback on performance in clinical practice.

AIS was used as an example in the present study, but similar funnel plots could be applied to evaluate performance on quality of care for other patients. Other common process measures involving time to treatment are waiting time from index event to carotid intervention in patients with symptomatic carotid stenosis, for which the norm is set at treatment within 14 days,^18^ and time between diagnosis and treatment of patients with cancer, for which a maximum of 5 weeks is set in the Netherlands.^19^ For both examples, earlier treatment could be beneficial, and targeting the whole distribution instead of using a cut-off value could substantially reduce time to treatment. This is also applicable to other process measures than time to treatment, such as glycated hemoglobin (HbA1c) level in diabetes care. Given that the median HbA1c is often reported as a performance measure, while quality initiatives are evaluated as percentage of patients achieving the target level, this might also have potential to direct quality improvement initiatives, especially as large variation between centres has been described.^20 21^

In conclusion, to provide more comprehensive feedback to hospitals concerning their performance on time to treatment, both types of funnel plots give additional information on whether performance should be improved for processes involving all patients or that patients at the extreme of the distribution, in this case patients with a substantial delay, should be investigated for potential explanatory factors to enable tailored improvement initiatives there. Using patients with AIS treated with IVT as an example, we showed that hospitals with high median DNT and hospitals with high proportions of patients with substantially delayed DNT are not necessarily the same. It could provide important additional information to hospitals that their median DNT was worse even without having more patients with substantially delayed DNT, which was the case for up to a quarter of the hospitals. This could give them the opportunities to improve further, even with the overall median DNT already being low in the Netherlands.
