# Supplementary material for: Enhancing feedback on performance measures: the difference in outlier detection using a binary versus continuous outcome funnel plot and implications for quality improvement
Source: BMJ Qual Saf. 2020 Feb 7;30(1):38–45. doi: 10.1136/bmjqs-2019-009929 (PMC7788228; doi:10.1136/bmjqs-2019-009929)
Supplement: Supplementary data [file bmjqs-2019-009929supp001.pdf]

Supplement 1

Permutation tests for funnel plots of door-to-needle time

Supplement to original article "Enchancing feedback on performance measures: the difference in outlier detection using a binary versus continuous funnel plot and implications for quality improvement."

Contents

Preliminaries	1
Funnel for exceeding the median	2
Funnel for exceeding the 90th percentile	3
Funnel for the median	4
Check	5
Funnel for the 90th percentile	6
Session information	7

Preliminaries

```
library(foreign)
data = read.csv(file.choose(), sep = ";")
data = data[!is.na(data$ DNT) & !is.na(data$ id),]
data$id = factor(data$ id)
set.seed (123)


DNT = data[,2 ]
m = median (DNT,na.rm=TRUE)
n = table(data [,1])
```

Door-to-needle time (DNT) has a skewed distribution, as shown in histogram below.

```
hist (DNT,1000)
```

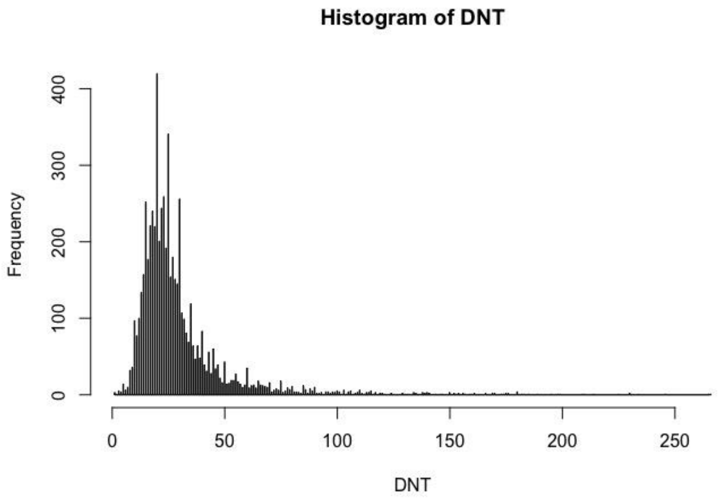

Funnel for exceeding the median

The median door-to-needle time is 24 minutes. We define a binary indicator as DNT > 24, and make a standard funnel plot.

```
O = unlist(tapply(DNT > 24, data[,1], sum))
p0 = mean(DNT > 24)
n = as.vector (n)
E = n*p0
plot(n, O/n, ylim = c(0,1), bty = “l”, ylab = ‘Observed proportion’, xlab = ‘Number of patients’, abline(h=p0))
nn = seq(0,1.1*max(n), max(n)/100)
L = (sqrt(nn*p0) – 1.96/2)^2/nn
L = pmax(0,L)
U = (sqrt(nn*p0) + 1.96/2)^2/nn
lines(nn, L, col = ‘red’)
lines(nn, U, col = ‘red’)
```

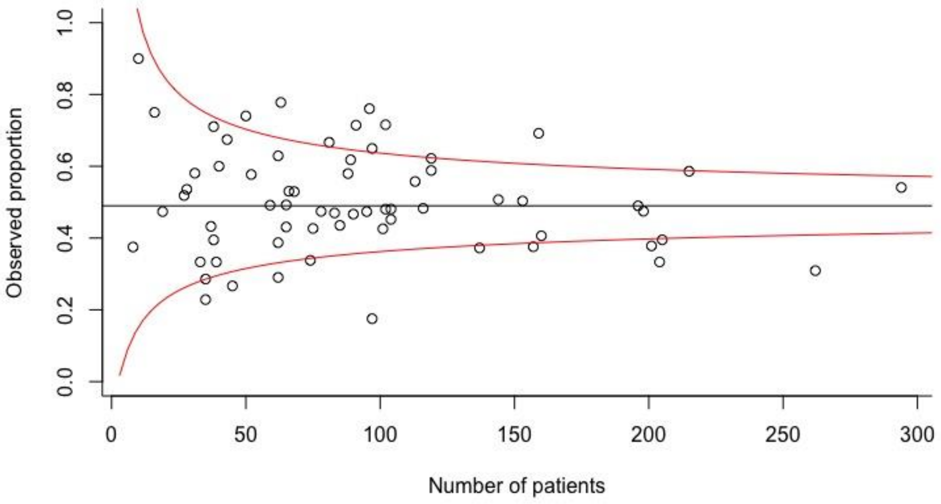

Funnel for exceeding the 90% percentile

The 90th percentile of the DNT is 50 minutes. We define a binary indicator as DNT > 50, and make a standard funnel plot.

```
O = unlist(tapply(DNT > 50, data[,1], sum))
p0 = mean(DNT > 50)
n = as.vector (n)
E = n*p0
plot(n, O/n, ylim = c(0,1), bty = "l", ylab = 'Observed proportion', xlab = 'Number of patients', abline(h=p0))
nn = seq(0,1.1*max(n), max(n)/100)
L = (sqrt(nn*p0) – 1.96/2)^2/nn
L = pmax(0,L)
U = (sqrt(nn*p0) + 1.96/2)^2/nn
lines(nn, L, col = 'red')
lines(nn, U, col = 'red')
```

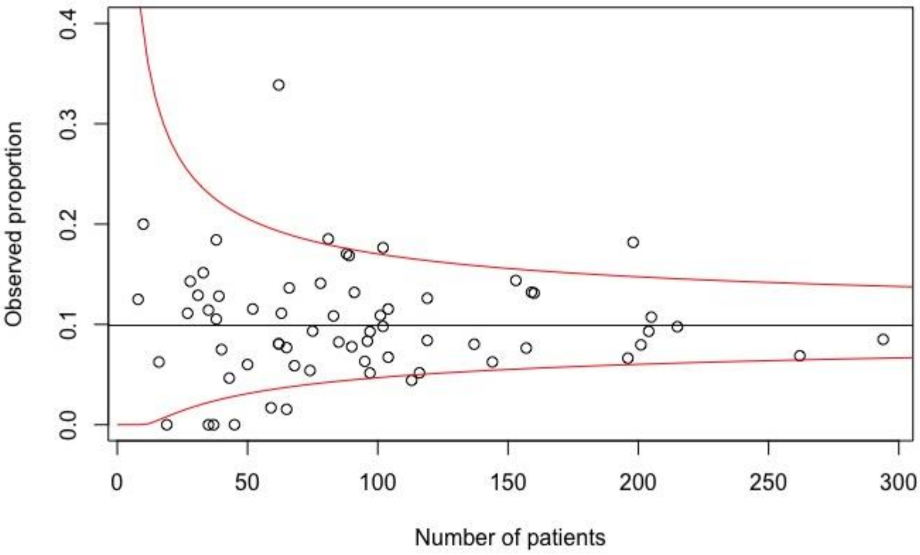

Funnel around the median

For varying numbers of patients, we compute the distribution of the median DNT by repeated random sampling from all available DNTs (B=10,000). We display the 2.5 and 97.5 percentiles of this distribution as control limits in the funnel plot. By checking if the median DNT of a particular hospital falls outside the control limits, we can test if there is significant deviation from the overall (nationwide) performance.

```
observed = unlist(tapply(data[,2 ], data[,1 ], median))          # observed medians
N = max(n)
nn = c(seq(2, 10, 2), seq(12,N, 10))
perm.id = rep(1:length(nn), nn)

perm.observed=NULL
for (i in 1:10^4 ){
        ind = unlist(mapply(sample, nn, x=nrow(data)))
        perm = data[ind,2 ]                                       # permute the data
        perm.observed = rbind(perm.observed, tapply(perm, perm.id, median))
}

L = apply(perm.observed,2 ,quantile,0.025 )
U = apply(perm.observed,2 ,quantile,0.975 )
plot (n, observed, bty="l", xlab = “Number of patients', ylab = “Median DNT (in minutes)')
abline (h = m)
Usmooth = -isoreg(nn, -U)$yf
Lsmooth = isoreg(nn, L)$yf
lines(nn, ceiling(Usmooth), type = "s", col = "red")
lines(nn, floor(Lsmooth), type = "s", col = "red")
```

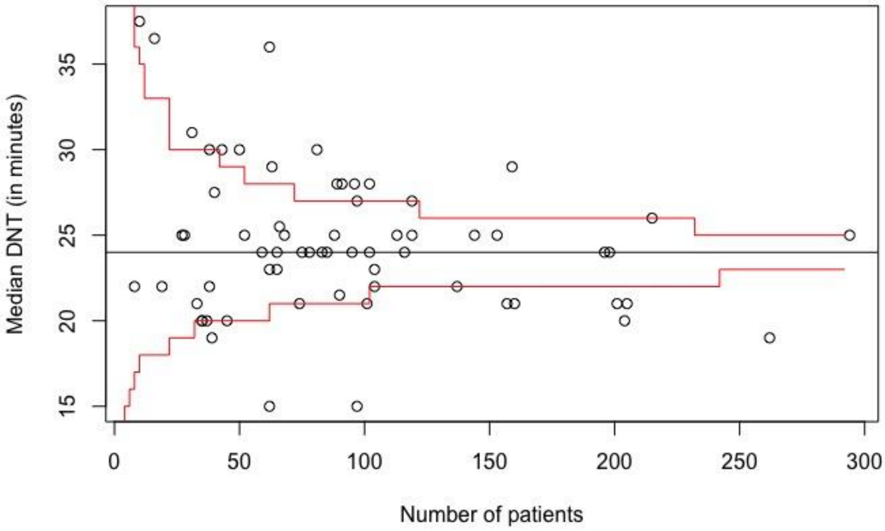

Note that in both funnel plots no adjustment for case mix factors is required for performance evaluation.

Check

If the DNTs at a particular hospital do not deviate from the overall (nationwide) performance, there should be 5% chance of falling outside the funnel plot around the median that we have constructed. We have checked this by randomly permuting the center identifiers (thus removing any center effects) and then re-plotting the median DNTs. As expected, we find that that now most (but not all) hospitals fall inside the funnel plot.

```
data[,1] =  data[sample(nrow(data)),  1]

observed =  unlist(tapply(data[,2], data[,1], median))
n = table(data[,1])
n = as.vector(n)
N= max(n)
nn = c(seq(2, 10, 2), seq(12, N, 10))
perm.id = rep(1:length(nn), nn)
perm.observed = NULL
for (i in 1:10^4) {
		ind = unlist(mapply(sample, nn, x=nrow(data)))
		perm = d[ind, 2]
		perm.observed=rbind(perm.observed, tapply(perm, perm.id, median))
}

L = apply(perm.observed, 2, quantile, 0.025)
U = apply(perm.observed, 2, quantile, 0.975)
plot (n,observed, xlab=”Number of patients”, ylab = “Median DNT (in minutes)”)
abline (h=m)
Usmooth = -isoreg(nn, -U)$yf
Lsmooth = isoreg(nn, L)$yf
lines(nn, ceiling(Usmooth), type ="s", col = "red")
lines(nn, floor(Lsmooth), type ="s", col = "red")
```

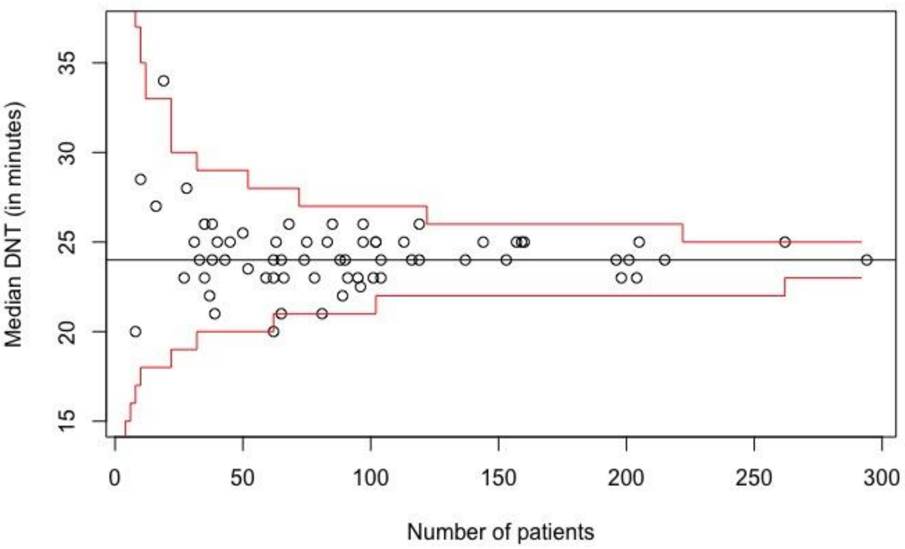

Funnel around the 90th percentile

Similar to the funnel plot around the median, we compute the distribution of the 90th percentile DNT by repeated random sampling from all available DNTs (B=10,000). We display the 2.5 and 97.5 percentiles of this distribution as control limits in the funnel plot.

```
P90 = function(x){
quantile(x,0.9)}
m = quantile(data[,2], 0.90)

observed=unlist(tapply(data[,2], data[,1], P90))                    # observed medians
N=max(n)
nn=c(seq(2,10,2),seq(12,N,10))
perm.id =rep(1:length(nn),nn)
perm.observed=NULL

for (i in 1:10^4){
        ind=unlist(mapply(sample, nn, x=nrow(data)))
        perm=data[ind,2]                                            # permute the data
        perm.observed=rbind(perm.observed,tapply(perm, perm.id, P90))
        }
L=apply(perm.observed,2,quantile,0.025)
U=apply(perm.observed,2,quantile,0.975)

plot(n, observed, bty="l", xlab = 'Number of patients', ylab = '90% percentile DNT')
abline(h = m)
Usmooth = -isoreg(nn, -U)$yf
Lsmooth= isoreg(nn, L)$yf
lines(nn, ceiling(Usmooth), type = 's', col = 'red')
lines(nn, floor(Lsmooth), type = 's', col = 'red')
```

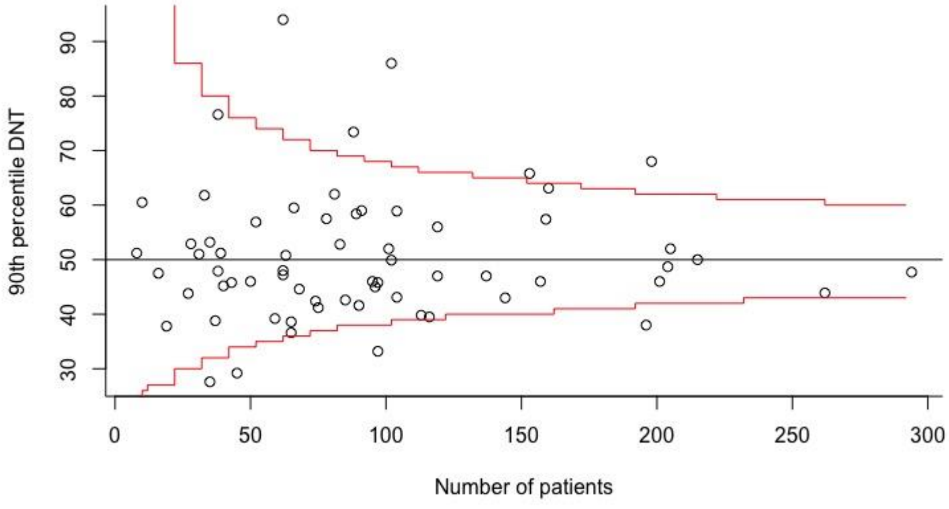

Session information

```
sessionInfo()
```

```
## R version 3.4.3 (2017-11-30)
## Platform: x86_64-apple-darwin15.6.0 (64-bit)
## Running under: macOS Sierra 10.12.6

## Matrix products: default

## locale:
## [1] nl_NL.UTF-8/nl_NL.UTF-8/nl_NL.UTF-8/C/nl_NL.UTF-8/nl_NL.UTF-8

##attached base packages:
## [1] stats     graphics  grDevices utils     datasets  methods   base

## other attached packages:
##  [1] cowplot_0.9.4   car_2.1-6       forcats_0.2.0   stringr_1.2.0   purrr_0.2.4     readr_1.1.1     tidyr_0.7.2
##  [8] tibble_2.0.1    tidyverse_1.2.1 lubridate_1.7.3 sjPlot_2.6.2    ggplot2_2.2.1   dplyr_0.8.0.1
```
